# Hypertrichosis

**Published:** 1899-03

**Authors:** 


### Hypertrichosis.

There are few chronic diseases that give rise to more real discomfort than this cosmetic defect. Numbers of doctors have almost piteous appeals from female patients on whom the development of a hirsute facial appendage is a source of as much worriment as it would be of joy to their young male relatives. So many different methods have been employed for its removal in the past, and so many exaggerated claims made for each new method, and yet recurrence has been the rule, that the ordinary general practitioner is apt to doubt that there is really any effective lasting method of depilation, and so advises his patients against attempts at relief.

The electrolytic method of removing the superfluous hairs of Trichiasis,—the invention and practical development of which, by the way, we owe entirely to Americans,—has been now before the profession nearly a quarter of a century. It has been generally adopted in Europe, and especially in Paris is used extensively and with the best satisfaction. "The question is often asked," says Dr.

Jackson, in his Manual of Skin Diseases.* "is the removal of the hair by this method permanent?" This question may be answered: "It is, without a shadow of a doubt." The answer has the advantage of being definitely decisive, something that is not always characteristic of therapeutic suggestions, especially in skin diseases. With the refinements in the use of the electrolytic needle that twenty-five years of practical experience with it has given, the depilation is now almost invariably successful from the beginning, and a new growth of hair afterwards is an anomalous irritative hyperplasia which is extremely rare, or a sign of failure to destroy the hair bulbs completely at first. The danger of scarring is also reduced to a minimum, and with reasonable care the ciccatrization will never be more than the minutest points on the skin, and seldom will be noticeable at all. There would really seem to be very little reason any more for sensitive people to suffer the discomfort they usually do because of the persistent presence of this unedsirable hirsute adornment.

*From advance sheets of the third edition of "Jackson on Diseases of the Skin." Lea Brothers & Co., publishers.

